# Enhancement of grain number per spike by RNA interference of cytokinin oxidase 2 gene in bread wheat

**DOI:** 10.1186/s41065-018-0071-7

**Published:** 2018-10-02

**Authors:** Yulian Li, Guoqi Song, Jie Gao, Shujuan Zhang, Rongzhi Zhang, Wei Li, Mingli Chen, Min Liu, Xianchun Xia, Thierry Risacher, Genying Li

**Affiliations:** 1 Crop Research Institute, Shandong Academy of Agricultural Sciences; Key Laboratory of Wheat Biology and Genetic Improvement on North Yellow and Huai River Valley, Ministry of Agriculture, National Engineering Laboratory for Wheat and Maize, Jinan, 250100 Shandong China; 2 Biogemma, Site de la Garenne, CS 90126, 63720 Chappes, France; 3 Institute of Crop Sciences, National Wheat Improvement Center, Chinese Academy of Agricultural Sciences (CAAS), 12 Zhongguancun South Street, Beijing, 100081 China

**Keywords:** *CKX2.4*, Grain number per spike, Transgenic plant, *Triticum aestivum*

## Abstract

**Background:**

This study aimed to validate the function of CKX gene on grain numbers in wheat.

**Methods:**

we constructed and transformed a RNA interference expression vector of *TaCKX2.4* in bread wheat line NB1. Southern blotting analysis was used to select transgenic plants with single copy. The expression of *TaCKX2.4* gene was estimated by Quantitative real-time PCR (qRT-PCR) analysis. Finally, the relation between expression of *TaCKX2.4* gene and grain numbers was validated.

**Results:**

Totally, 20 positive independent events were obtained. Homozygous lines from 5 events with a single copy of transformed gene each were selected to evaluate the expression of *TaCKX2.4* and grain numbers per spike in T_3_ generation. Compared with the control NB1, the average grain numbers per spike significantly increased by 12.6%, 8.3%, 6.5% and 5.8% in the T_3_ lines JW39-3A, JW1-2B, JW1-1A and JW5-1A, respectively.

**Conclusion:**

Our study indicated that the expression level of *TaCKX2.4* was negatively correlated with the grain number per spike, indicating that the reduced expression of *TaCKX2.4* increased grain numbers per spike in wheat.

## Background

The plant hormone cytokinin (CK) promotes cell proliferation and differentiation, and regulates plant growth and development from many processes, such as senescence, shoot and root balance, transduction of nutritional signals, leaf senescence, chloroplast formation and crop productivity [1–3]. CK in grains during the early stage of grain development play an important role in regulating grain filling pattern and consequently influence grain filling percentage [4], and CK in the grains may mediate cell division in rice endosperm at early grain filling stages [5]. CK are the most potent general coordinator between the stay-green trait and senescence. Stay-green can not only increase the yield of wheat but also its resistance to heat stress during active photosynthesis [6]. Exogenous cytokinins can sustain longer active photosynthetic period during the grain filling stage, transfer more assimilates to the grain [7], and increase grain yield under heat stress [6]. The increase of photosynthetic productivity can lead to high yields through improvement of leaf anatomical and biochemical traits including tolerance to non-optimal temperature conditions [8].

Cytokinin oxidases/dehydrogenases (CKX) are important in controlling local cytokinin levels and contribute to the regulation of cytokinin-dependent processes [9]. CKX is regarded as a negative regulator of cytokinin [10]. Overexpression of *CKX* gene led to reduced endogenous cytokinin contents in plants [11].

A number of *CKX* genes were cloned from *Zea mays* [12], *Arabidopsis* [13], orchid [14], rice [15], barley and wheat [16–20]. The functions of CKX genes were demonstrated in some plants by transgenic technology. The relationship between CKX and CK has been extensively investigated to understand their functions in plant development. The CKX activity may be induced by the levels of CK [20]. In *Arabidopsis*, the transgenic plants with an overexpression of *AtCKX1* and *AtCKX3* had fewer flowers on each inflorescence stem, indicating a reduction in the ability of apical inflorescence meristems to form new flower primordia [13]. In rice, the transgenic plants with low expression level of *OsCKX2* had an increased grain numbers, whereas the transgenic plants with a higher expression level of *OsCKX2* showed a lower grain numbers. In addition, an *OsCKX2* null variety had high grain yield [15]. This suggested that the different transcript levels of *OsCKX2* were responsible for the phenotypic differences of these rice varieties [15]. Down-regulation of *OsCKX2* expression increased tiller numbers and improved grain weight [21]. Overexpression or suppression of *GhCKX* (*Gossypium hirsutum* L.) in transgenic tobacco led to deficiency of cytokinin (e.g., fewer or no flowers) or over-production of cytokinin (e.g., more flowers and capsules) [19]. In addition, silencing of *HvCKX1* in transgenic barley resulted in a lower CKX activity and a higher grain yield [17]. So far, there have been no reports of transgenic wheat to prove the function of *TaCKX* gene.

Wheat is one of the most important staple crops worldwide. To study the relationship between the expression level of CKX and wheat productivity, Zhang et al. [19] cloned *TaCKX6* and observed that its haplotype variants were significantly associated with the 1000-grain weight based on linkage mapping, association study and gene expression analysis. The expression level of two *TaCKX2* genes was significantly correlated with grain numbers per spike [18]. However, the relationship between the *CKX* gene expression and number of reproductive organs in transgenic plants remains unknown. In the present study, a *TaCKX2.4* [22] RNA interference vector (piCKX2.4) was constructed and transformed into immature embryos using the seed inoculation method to validate the function of *TaCKX2.4* and the relationship between *TaCKX2.4* and grain numbers in bread wheat.

## Materials and methods

### Plant material and preparation of explants

Stock plants of NB1 were produced in 17 cm diameter pots, four plants per pot. Plants are grown in KLASMANN compost, a thin layer of medium vermiculite is scattered on top of the compost. Aquamat was used under the pots to keep the moisture. Place pots in a greenhouse at 25 °C day/20 °C night with 16 h photoperiod, 500 μE/m 2 /s.

### Construction of RNAi interference vector

**Fig. 1:**
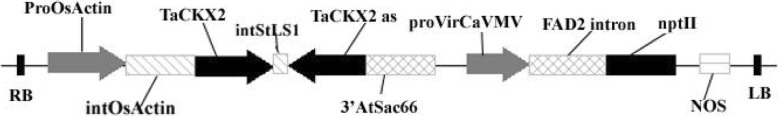
T-DNA region of the plasmid pBIOS2043-TaCKX2

In order to use hairpin construct to generate a higher efficiency of RNA silencing, we selected the conserved domain of *TaCKX2.4* (located on chromosome 3A) as the RNAi target sequence. The conserved fragment of 1–495 bp of *TaCKX2.4* (JN381555.1) was used to design RNAi hairpin structure. The conserved hairpin was inserted into pBIOS2043 under the regulation of the promoter OsActin 1 and Act 1 intron 1 [23] to construct the plant expression RNAi vector pBIOS2043-*TaCKX2.4* (Fig.1).

### Bacterial strains and culture conditions

*Agrobacterium tumefaciens* strain EHA105 was used for gene transformation. Agrobacterium cells were cultured on YEP plate with appropriate antibiotic (Kanamycin, 50 mg/L) at 28 °C for 1 d. Then the bacteria was collected, and re-suspended in 3 ml TSIM medium (MS medium 4.41 g/l, Myo-inositol 100 mg/l, MES 50 mg/l, Glucose 10 g/l) with 200 μM Acetosyringone (AS), and the bacterial suspension was transferred into a small universal tube.

### Plant transformation

Wheat immature embryos transformation was performed as described in Risacher et al. [24]. one microlitre bacterial suspension was injected into each immature seed on the spike using a syringe. The injection should be done between the endosperm and the scutellum. After all ears were inoculated, fix a support cane outside the cylinder and cover with a translucent plastic bag sealed at the base. After 2 d co-cultivation at 22 °C, isolate the inoculated seeds and extract the embryos by tweezers and blade, and plate the embryos on W4 rest medium (containing MS medium, 100 mg/l Myo-inositol, 50 mg/l MES and 10 g/l glucose) at 28.5 °C/23 °C (16 h/8 h) for 5 d. In 12 d after isolation, the calli were transferred to the selection medium (W4 added 2.5 ml/l geneticin) at 25.5 °C/23 °C (16 h/8 h), and 2 weeks later, the calli were cut into pieces and transferred to fresh selection medium. After 2 weeks, the calli were transferred to regeneration medium. Regenerated shoots were separated from the calli and transferred onto rooting medium containing MS salts (20 g/l sucrose, 6 g/l agarose, 2.5 ml/l Geneticin and 2 ml/l Kinetin) [24]. Rooted shoots were transplanted into soil and cultured in the greenhouse. The media for calli of controls didn’t have geneticin.

### Preliminary screening of homozygous transgenic plants with single copy

Thirty T_1_ generation seeds harvested from each T_0_ transgenic plant were selected randomly and put into 10 ml tubes. Two times volumes of 2 g/l Kanamycin solution were added into the tubes. After being treated at room temperature for 24 h, the seeds were put on the vermiculite to grow at 25 °C. The normal plants from the lines with the ratio of 3 normal to 1 albino plants were selected and transferred them to the soil, grew in the greenhouse with controlled environment at 25 °C/20 °C (16 h/8 h). The seeds of T_2_ generations were harvested in each individual plant. The T_2_ generation seeds were treated by Kanamycin according to the method mentioned above [24]. The lines with all normal plants, derived from each T_1_ plant, should have a single copy. The transgenic lines with a single copy were verified by Southern blotting analysis.

### Molecular analysis of transformed plants

#### PCR analysis

Transgenic plants were selected by Polymerase Chain Reaction (PCR). Genomic DNA was extracted from young leaf tissues of receptor NB1 and transgenic plants using plant genomic DNA Extraction Kit (Tiangen Biotech, Beijing, China). The gene-specific primers (5’-ATTCTTATTTCTTTCCAGTAGC and 5’-AGAAGCGGCATAATGTGAGA) were used to amplify an 882-bp fragment of FAD2 intron, a fragment of the interference vector. PCR was performed in a total volume of 20 μl containing 50 ng of genomic DNA, 2 μl PCR buffer, 0.2 mM of forward and reverse primers, and 1.5 U of *Taq* polymerase using a PCR Reaction Kit (Tiangen Biotech, Beijing, China). The PCR reaction included DNA denaturation at 94 °C for 5 min, followed by 38 cycles of denaturation at 94 °C for 30 s, annealing at 55 °C for 30 s, and extension at 72 °C for 30 s, and a final extension at 72 °C for 10 min. Only the positive transgenic plants can be amplified the expected size, because the FAD2 was the fragment of the vector. In order to confirm the band, we sequenced the amplified product.

#### Southern blotting analysis

Genomic DNA was extracted from leaves of T3 transgenic plants with single copies selected preliminarily by Kana and non-transgenic plants using plant genomic DNA Extraction Kit (Tiangen Biotech, Beijing, China). About 20 μg of DNA was successfully digested with 5 U of EcoRV and incubated at 37 °C for 24 h. The digested genomic DNA fragments were separated on a 0.8% (*w*/*v*) agarose gel, and transferred onto Zeta-Probe GT nylon membrane (Bio-Rad, Hercules, CA, USA). The DNA fragments were fixed to the membrane by UV cross linking. The 882-bp PCR fragment of FAD2 intron was labeled with digoxin. The probe labeling, hybridization, washing and detection procedures were performed according to instructions of the DIG High prime DNA labeling and Detection Starter KitII (Roche, Mannheim, Germany).

#### Quantitative real-time PCR (qRT-PCR) analysis

RNA was extracted from leaves of transgenic plants and the control NB1 using TRIZOL Reagent (Invitrogen, CA, USA) and reverse transcription of RNA was performed using M-MLV RTase Synthesis Kit (Takara, Japan). Quantitative real-time PCR (qRT-PCR) was performed using the Rotor-Gene 3000 Series real time DNA amplification system under the following conditions: 95 °C for 10 min; 40 cycles of 95 °C for 10 s, 60 °C for 20 s and 72 °C for 20 s. Melting curve analysis was performed at 95 °C for 15 s, 60 °C for 1 min, and 97 °C, continuous, using the *TaCKX2.4*-specific primers (5’-TGTCGGTCGAAGGCCAGTA and 5’-TAGTCGGTCCACGA ACGC). Melting curve analysis was included to verify the specificity of DNA amplification. Expression of the reference gene tubulin, served as quantifying control, was monitored using gene-specific forward and reverse primers (5’-AACTTCGCCCGTGGTCAT and 5’-CAGCGTTGAATACAAGGAATC) under the same condition as that of *TaCKX2.4*. Three biological replicates were performed for each line and three technical replicates were analyzed for each biological replicate.

#### Agronomic traits of T_3_ transgenic plants

Five single-copy transgenic lines and the control NB1 were grown in the isolated field for genetically modified organism (GMO) in Shandong Academy of Agricultural Sciences, Shandong, China. Field trials were conducted in completely randomized blocks with three replicates. Each plot consisted of four 4-m rows with 30 cm between rows and 20 cm between plants. The data for spike length (SL), spikelet number per spike (SPN) and grain number per spike (GN) were measured from the main spikes of ten randomly selected plants each plot. SL was the length from the neck node to the top of the spike (did not include the length of awn). Thousand-grain weight (TGW) was measured as the average weight of two independent samples of 1000 grains. The kernel length and kernel width were recorded for 20 randomly selected kernels from each line in triplicate, and the average value was used for subsequent analysis [25].

## Results

### Wheat transformation and PCR analysis

**Fig. 2:**
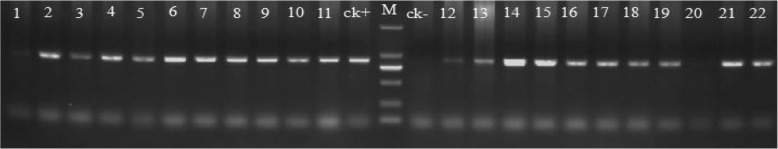
PCR detection of 22 putative transgenic plants. 1~ 22 are putative transgenic plants, positive control (plasmid DNA as template, CK+) and negative control (non-transgenic plants, CK-)

The *TaCKX2.4* RNA interference vector (PiCKX2.4) was transformed into immature embryos of wheat line ‘NB1’ by *Agrobacterium*. There were 22 putative transgenic plants from 22 independent transgenic lines. In order to avoid the interference of endogenous genes in wheat, the presence of PiCKX2.4 vector in the putative transgenic plants was determined by genomic DNA PCR with the primers specific to FAD2 intron, a fragment of the interference vector. Twenty of 22 primary transformants that could amplify an 882-bp targeting band were PCR-positive (Fig.2), whereas the negative control had no amplified fragments.

### Southern analysis of T_0_ transgenic wheat and homozygous transgenic plants with single copy

**Fig. 3:**
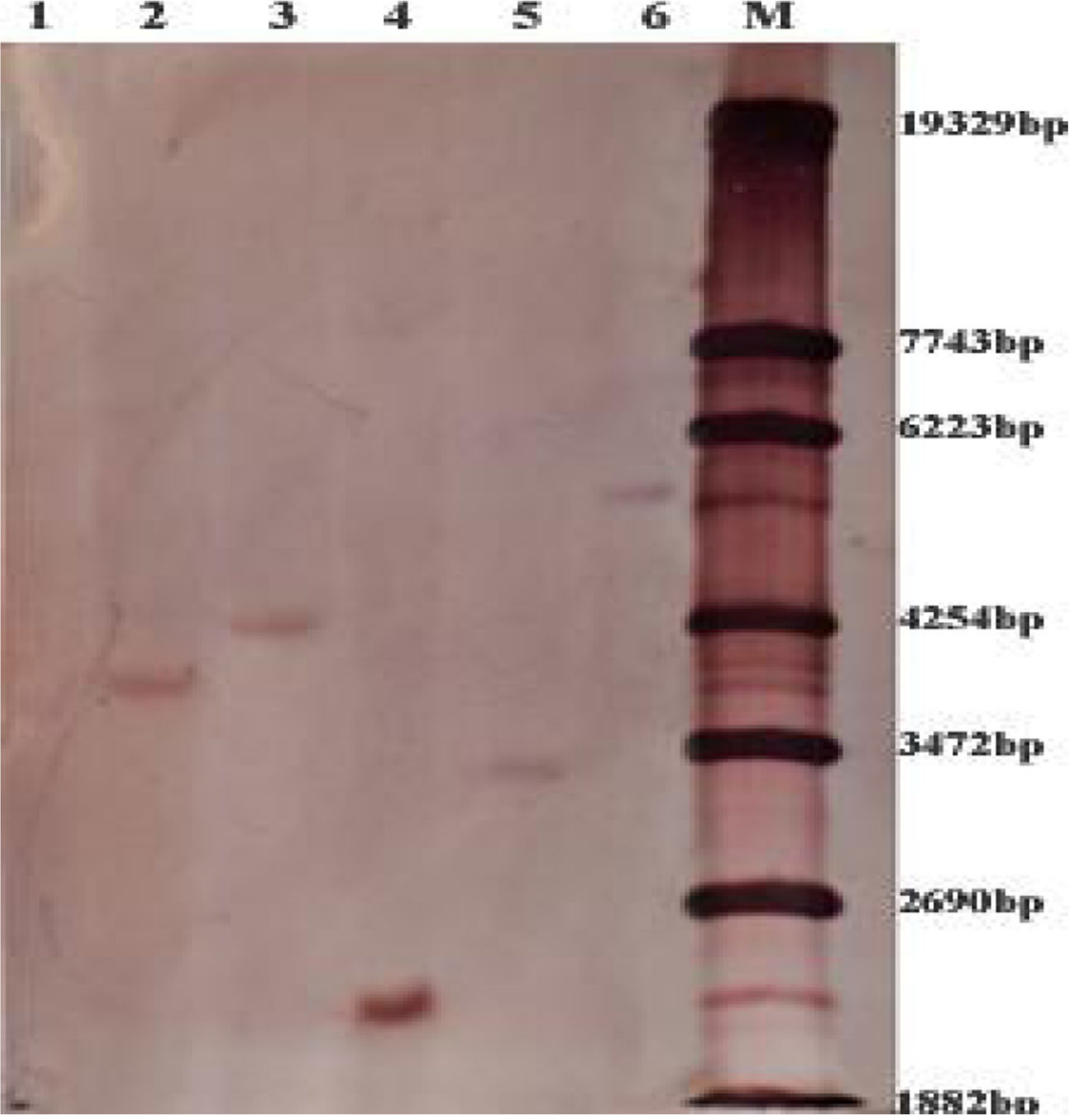
Southern blotting analysis in selected T_0_ primary wheat transformants. Genomic DNA digested with *Eco*R V and hybridized with FAD2 probe. The number of reactive bands in each lane represents the transgene copies in each transgenic line. Lane 1 is WT (NB1), lanes 2~ 6 are JW1-1A, JW5-1A, JW41-1B, JW39-3A and JW1-2B, respectively, M is the marker: λ-EcoT14 I digest (TaKaRa, Dalian, China)

Based on the preliminary results of PCR analysis, representative T_0_ transgenic lines were selected for gene integration analysis. Southern blotting analysis indicated that the FAD2 intron on the T-DNA were integrated into the wheat genome at single copies in the JW41-1B, JW1-2B, JW1-1A, JW5-1A and JW39-3A transgenic lines (Fig. 3).

In order to obtain homozygous transgenic plants with a single copy, 30 seeds from per T_0_ transgenic plant with a single copy were treated by 2 g/L Kana solution. The results showed that five lines had a 3:1 segregation of normal to albino plants, confirming that the five T_0_ transgenic plants were with single copy. Thirty seeds from per T_1_ normal line were treated by 2 g/L Kanamycin solution. Five lines with no segregation were homozygous and selected for subsequent analysis. PCR for T_1_ to T_3_ transgenic lines showed that PiCKX2.4 was stably inherited.

### *TaCKX2.4* expression in transgenic wheat with single copy

**Fig. 4:**
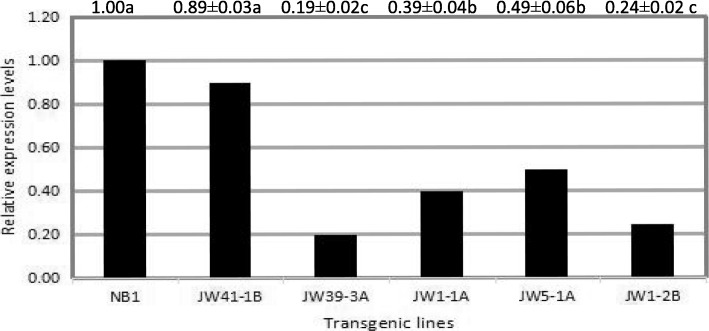
Relative expression of *TaCKX2* in wild type (NB1) and selected T_3_ transgenic lines by qRT-PCR. Total RNA was isolated from young leaves of NB1 and selected T_3_ transgenic lines. Different lowercase letters following the values indicate significant differences (*P* < 0.05)

The expression of *TaCKX2.4* in leaves of T_3_ lines from selected primary transformants with single copy was analyzed by qRT-PCR using specific primers. There was variation in the expression level of *TaCKX2.4* among the five selected transformants. In general, the *TaCKX2.4* transcript levels were much lower in the transformants than in controls (Fig. 4). Among them, the lines JW39-3A and JW1-2B had the lowest expression of *TaCKX2.4*, 0.19 and 0.24, respectively, relative to the control. Only the line JW41-1B had no significantly different expression from that of the control, which could be attributed to the low expression of shRNA-CKX2.4 in this transgenic line. Overall, the shRNA-CKX2.4 introduced into the wheat genome resulted in reduced expression of *TaCKX2.4* in these transgenic lines.

### The relationship between *TaCKX2.4* expression and wheat agronomic traits

**Fig. 5:**
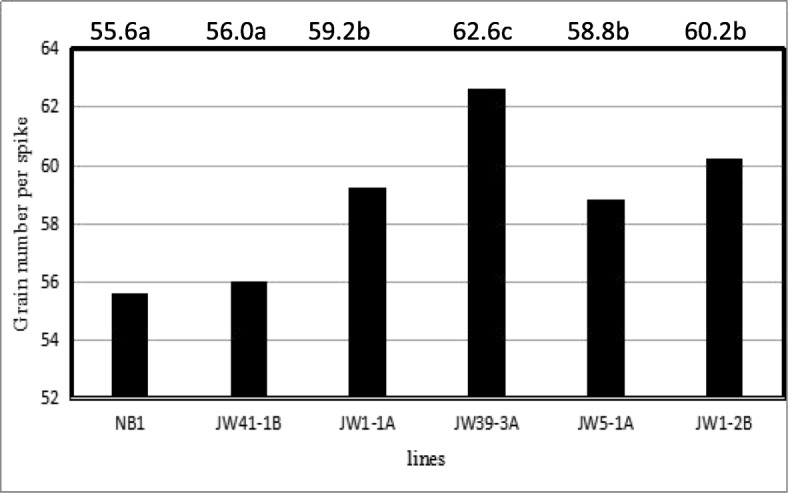
Grain numbers per spike from main tiller in NB1 and five transgenic lines. Different lowercase letters following the values indicate significant differences (*P* < 0.05)

The T_3_ homozygous seeds with single copy were grown in the field for phenotype observation. The field trials showed no significant differences in spike length, number of spikelet per spike, kernel length, kernel width and TGW between transgenic and control plants. However, the grain numbers per spike from main tiller of transgenic plants were significantly (*P* < 0.05) increased by 5.8–12.6% on average compared with those of non-transformed plants (Table 1 and Fig. 5).

**Table 1:** Comparison of phenotypes between transgenic lines and the control NB1

Line	Spike length (cm)	Kernel width (mm)	Kernel length (mm)	Thousand-grain weight (g)	Grain numbers per spike	Increased grain numbers per spike (%)
JW41-1B	10.9 a	3.40a	6.90 a	31.1 a	56.0 a	–
JW1-1A	11.0 a	3.35a	6.75a	29.5 a	59.2 b	6.5
JW39-3A	11.6 a	3.40 a	7.00 a	30.6 a	62.6 c	12.6
JW5-1A	10.7 a	3.45a	6.75 a	29.1 a	58.8 b	5.8
JW1-2B	11.1 a	3.45 a	6.75 a	29.1 a	60.2 b	8.3
NB1	10.8 a	3.40 a	7.00 a	30.9 a	55.6 a	–

Different lowercase letters following the values indicate significant differences (*P* < 0.05)

**Fig. 6:**
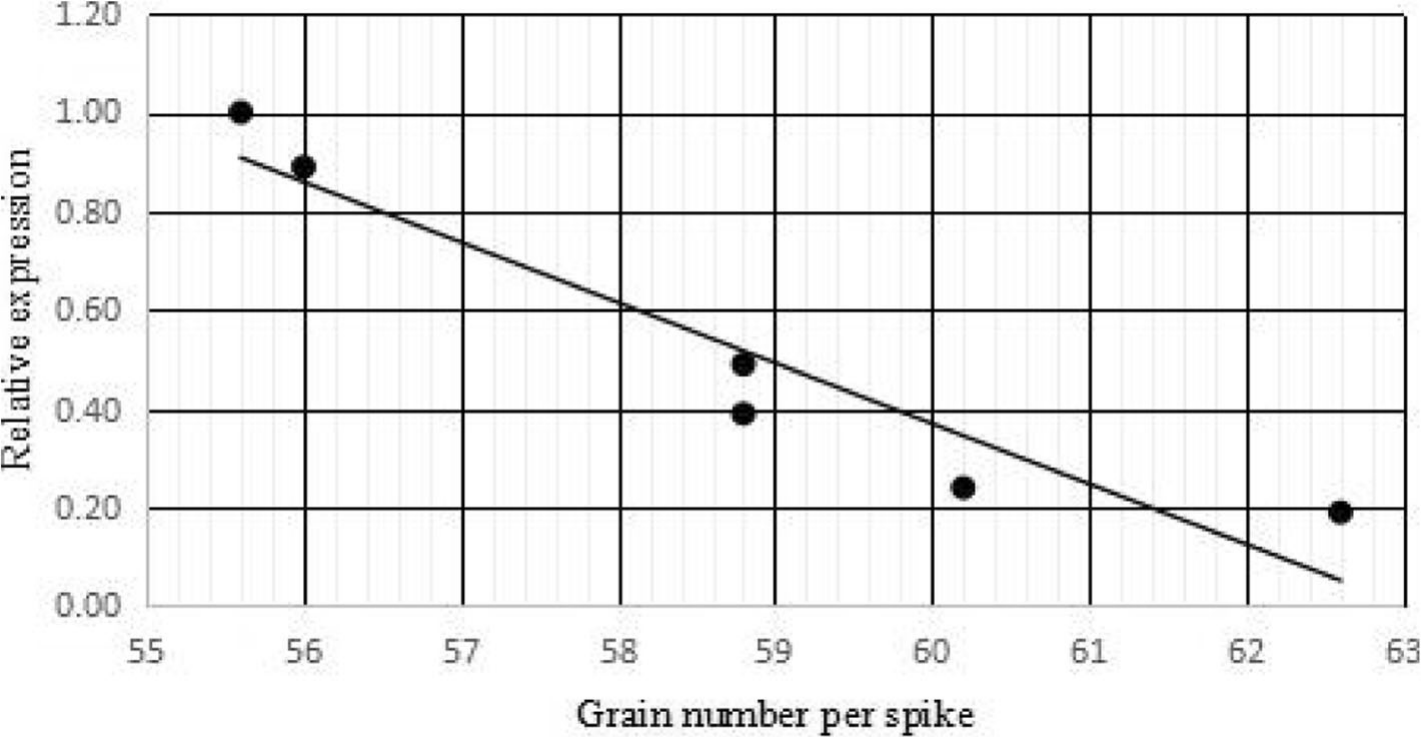
The trendline for the relation between relative expression of *TaCKX2* and grain numbers per spike from main tiller in NB1, JW41-1B, JW5-1A, JW1-1A, JW1-2B and JW39-3A

To evaluate the relationship between the expression level of *TaCKX2.4* gene and grain numbers per spike. qRT-PCR with RNA from the young spikes of five transgenic plants with single copy and control NB1 was performed. The expression of *TaCKX2.4* in the five transgenic plants was lower than that in the control (Fig. 4). The expression level of *TaCKX2.4* was negatively correlated with grain numbers per spike, indicating that the reduced expression of *TaCKX2.4* increased grain numbers in wheat (Fig. 6).

## Discussion

The aim of the current study was to specifically down regulate the expression of *TaCKX2.4* using shRNA-mediated gene silencing to examine its influence on wheat yield-related traits. Here we showed that iRNA-*TaCKX2.4* transgenic wheat lines expressed the shRNA efficiently and specifically reduced the expression of homologous *TaCKX2.4* (Fig. 4), consistent with those of transgenic rice and barley plants that harbored hairpin (hp) RNA-expression constructs containing rice *OsCKX2* sequences [21], and barley yellow dwarf virus-PAV sequences [26], and partial cDNA fragments of *HvCKX1* [17], resulting in blockage of the translation of target mRNAs into proteins. Our results showed that PiCKX2.4 was stably inherited in transgenic lines. The iRNA-*TaCKX2.4* transgenic lines in this study contained one copy of the silencing genes in the genome (Fig. 3), conferring an efficient reduction of *TaCKX2.4* transcript in all lines except for JW41-1B (Fig. 4).

Because CKX is the only known enzyme responsible for irreversible degradation of cytokinins in plants, mutant or transgenic plants with low expressing levels of *CKX* genes have been shown to possess a clear cytokinin-overproducing phenotype. Recent genetic transformation studies suggest that crop productivity can be enhanced by decreasing the expression of *CKX* gene [21]. Decreased expression of *OsCKX2* in transgenic rice by anti-sense also leads to increased grain numbers per panicle [15]. There were more capsules in transgenic CKX-suppression tobacco plants than in control [27].

In wheat, the relationship between the expression of *CKX* gene and the number of reproductive organs has not been confirmed by a direct validation via gene transformation before. Here we investigated the relationship between the expression level of *TaCKX2.4* gene and the grain number per spike, spike length, thousand-grain weight, seed length and seed width in transgenic wheat plants with *TaCKX2.4* RNA interference vector. The results showed a significantly negative correlation between the expression of *TaCKX2.4* and grain numbers, consistent with those in rice [21] and *Arabidopsis thaliana* [13], but opposite to previous results obtained in wheat where the increasing of *TaCKX2.1*/*TaCKX2.2* expression could lead to the increase of grain number per spike [18]. Decreased expression of the *TaCKX1*, *TaCKX2* and *TaCKX6* may lead to the accumulation of CK in NIL31 (*TaGW2-6A* allelic variants) [28]. The *TaCKX2.4* RNA interference vector reduced the activity of the cytokinin oxidase in transgenic plants, which caused accumulation of cytokinin in inflorescence meristems and increased the number of reproductive organs, leading to increasing grain numbers per spike.

In wheat,five *TaCKX2* genes were reported, and they were located on the short arms of the group 3 chromosome [18, 22]. *TaCKX2.4* was mapped on chromosome 3A, *TaCKX2.5* was mapped on chromosome 3B, and *TaCKX2.1*, *TaCKX2.2* and *TaCKX2.3* were mapped on chromosome 3D [18, 22]. Alignment of the CDS and amino acid of *TaCKX2.4* with *TaCKX2.1*, *TaCKX2.2*, *TaCKX2.3* and *TaCKX2.5* showed sequence identity of 93%, 93%, 85% and 85%, respectively, and 92%, 91%, 80% and 80%, respectively. High sequence identity indicates that they have similar biological functions [18].

To the best of our knowledge, this is the first study to verify the function of *TaCKX2.4* in wheat, where an efficient suppression of *TaCKX2.4* is successfully achieved by shRNAi.

## Conclusion

This study constructed a plant expression RNAi vector pBIOS2043-*TaCKX2.4* and obtained five transgenic wheat lines with single copies of transformed targeting gene using the infection of *Agrobacterium tumefaciens* strain EHA105. The positive transgenic plants had significantly lower expression of *TaCKX2.4* and higher grain numbers per spike than the control NB1. It was shown that the reduced expression of *TaCKX2.4* significantly increased grain numbers in wheat.

## Abbreviations

AS: Acetosyringone
CK: Cytokinin
CKX: Cytokinin oxidases/dehydrogenases
GMO: Genetically modified organism
GN: Grain number per spike
qRT-PCR: Quantitative real-time PCR
SL: Spike length
SPN: Spikelet number per spike
TGW: Thousand-grain weight
